# Caching mechanisms for habit formation in Active Inference

**DOI:** 10.1016/j.neucom.2019.05.083

**Published:** 2019-09-24

**Authors:** D. Maisto, K. Friston, G. Pezzulo

**Affiliations:** a Institute for High Performance Computing and Networking, National Research Council, Via P. Castellino, 111, Naples 80131, Italy; b The Wellcome Trust Centre for Neuroimaging, Institute of Neurology, University College London, London, UK; c Institute of Cognitive Sciences and Technologies, National Research Council, Via San Martino della Battaglia 44, Rome 00185, Italy

**Keywords:** Deliberative control, Habitual control, Habitisation, Active Inference, Caching

## Abstract

A popular distinction in the human and animal learning literature is between deliberate (or willed) and habitual (or automatic) modes of control. Extensive evidence indicates that, after sufficient learning, living organisms develop behavioural habits that permit them saving computational resources. Furthermore, humans and other animals are able to transfer control from deliberate to habitual modes (and vice versa), trading off efficiently flexibility and parsimony – an ability that is currently unparalleled by artificial control systems. Here, we discuss a computational implementation of habit formation, and the transfer of control from deliberate to habitual modes (and vice versa) within Active Inference: a computational framework that merges aspects of cybernetic theory and of Bayesian inference. To model habit formation, we endow an Active Inference agent with a mechanism to “cache” (or memorize) policy probabilities from previous trials, and reuse them to skip – in part or in full – the inferential steps of deliberative processing. We exploit the fact that the relative quality of policies, conditioned upon hidden states, is constant over trials; provided that contingencies and prior preferences do not change. This means the only quantity that can change policy selection is the prior distribution over the initial state – where this prior is based upon the posterior beliefs from previous trials. Thus, an agent that caches the quality (or the probability) of policies can safely reuse cached values to save on cognitive and computational resources – unless contingencies change. Our simulations illustrate the computational benefits, but also the limits, of three caching schemes under Active Inference. They suggest that key aspects of habitual behaviour – such as perseveration – can be explained in terms of caching policy probabilities. Furthermore, they suggest that there may be many kinds (or stages) of habitual behaviour, each associated with a different caching scheme; for example, caching associated or not associated with contextual estimation. These schemes are more or less impervious to contextual and contingency changes.

## 1 Introduction

Imagine a tourist visiting a new city for the first time, having to choose which bus or metro to take to reach the city centre. This choice problem requires planning and the careful consideration of alternative routes, with their respective costs and benefits. However, in successive visits to the same city, the tourist can skip these complicated evaluations and reuse good solutions – unless (for example) a metro station has been closed in the meantime, making some replanning necessary. This simple example illustrates the fact that humans and other animals can flexibly invest more or less resources (e.g., attention resources) into cognitive tasks, depending on task demands and uncertainty. A traditional distinction in human psychology and the animal learning literature is between deliberative (goal-directed) and habitual (automatic or routine) systems: deliberative, goal-directed decisions consider the current situation and use prediction to foresee the consequences of potential plans, while habits reflect information slowly accumulated over time; e.g., information about past rewards collected while executing a given action. In general, deliberative processing is considered more cognitively demanding but also more flexible than habits [3], [14], [47]. This distinction has received considerable empirical support but its computational principles and neuronal bases are still debated [15]. A particularly challenging question is the way an adaptive agent should balance declarative and habitual strategies and under which conditions it should allocate or transfer control between them. The ability to transfer control from (flexible but demanding) deliberative strategies to (cheaper but inflexible) habitual strategies, and vice versa, is considered a hallmark of adaptive behaviour and cognitive control, which permits one to combine adaptivity and parsimony; but its mechanisms are incompletely known.

### 1.1 Background and open questions

In this article, we ask how control should be transferred from deliberative to habitual control strategies, and vice versa, from the perspective of a normative – Active Inference – agent model [24].

The transfer of control from deliberative to habitual strategies (after a number of trials that is plausibly sufficient to reduce environmental uncertainty and behavioural variability) is called *habitisation* — and has been studied widely in the animal and human learning literature. While developing habits is generally useful to alleviate cognitive and behavioural demands, it can also have drawbacks. When an animal performs a behavioural (e.g., lever pressing) task for which it is rewarded (e.g., with a food) for a long period, and operates in conditions of low environmental volatility, it can develop habits that become inflexible. In other words, efficient habits preclude a context sensitivity that is necessary when environmental contingencies change. Behavioural inflexibility is assessed using a number of procedures, such as by testing the animal’s sensitivity to *reinforcer devaluation* (e.g., the food delivered by lever pressing is deprived of value because the animal is selectively satiated with the same food [3], [4], [9], [31], [32]). If the animal perseverates (e.g., continues pressing the lever) after the reinforcer devaluation, it is considered to be under habitual control and reflecting the loss of the ability to switch back to a more flexible, context-sensitive, deliberative (or goal-directed) form of control.

However, inflexible perseveration is a rather extreme situation that is usually associated with overtraining (or lesions [4], [31]): in most real-time situations, a person whose actions are controlled automatically is still able to re-engage deliberative processing when necessary, and especially when automatic control fails [47]. For example, a person who is learning to drive initially devotes her full attention to the driving task; but she can successively automatise most actions (and perform other tasks while driving). Crucially, if something goes wrong with the habitual policy (e.g., pressing the brake pedal produces a loud noise), the driver can re-engage her deliberative system and redeploy her attention to the task. This example suggests that in some cases, it is possible to transfer control from deliberative to habitual systems (and routinise behaviour), without losing the ability to transfer control back from habitual to deliberative systems – perhaps with the aid of an additional (supervisory) system that monitors the success of automatic strategies and/or contextual changes [47]. In sum, as the above discussion prompts a set of questions about what habits are and how they are formed in the first place; whether there are different (more or less severe) forms of habitization [3], [47]; and under which conditions habit formation implies behavioral inflexibility.

A widespread assumption in the literature is that deliberative and habitual strategies of choice may correspond to two different control schemes that operate in parallel and continuously compete for being selected [10]. These two control schemes would correspond to model-based and model-free controllers of reinforcement learning, respectively. These two controllers differ in that the former entails a form of prospective evaluation of future rewards, whereas the latter uses cached action values; but importantly, they are learned in parallel. However, an emerging alternative is that deliberative strategies are acquired first and scaffold the acquisition of habitual strategies. For example, habitual strategies may derive from a “compression” or “caching” of deliberative strategies (e.g., by chunking action sequences) that entail a more parsimonious use of cognitive resources while preserving accuracy – at least when the agent has no residual uncertainty about the environment [11], [12], [23], [49], [60], [61]. As we discuss below, the idea of augmenting a deliberative architecture with the ability to cache policies constitutes a promising approach to understand habit formation and their deployment under adequate contextual conditions.

### 1.2 Habit formation and transfer of control in Active Inference

In this article, we address the above questions from the perspective of an artificial (Active Inference) agent that deploys adaptive control in simulated foraging scenarios [26], [53]. The active inference agent is quintessentially deliberative; but here we explore how it can be endowed with the abilities to acquire habits and to transfer control from (more demanding) deliberative planning strategies to (less demanding) habitual routines by caching policies, when the situation permits; for example, when the choice context is stable. On this perspective, when the current choice situation induces no residual uncertainty or risk, an agent can select a behavioural policy act based on a learned (cached) score, rather than engage in a full deliberative (policy evaluation and selection) process. The underlying idea is that, if contingencies do not change, policy scores are stable and *caching* them might be more efficient than re-calculating them again.

Our simulations offer a novel perspective on the relationship between deliberation and habits, by suggesting that habitual policies can form and be selected by *caching* deliberative policies – and that this saves resources (e.g., computational time). The transfer of control from deliberative to habitual control is based on a simple (threshold-based) evaluation of habitual policy accuracy under the current context, whereas the opposite transfer from habitual to deliberative control depends on a mechanism that estimates contextual changes. In other words, an agent that is under the control of a habitual policy can transfer control to deliberative processes as long as it can recognize that the context has changed; conversely, failing to notice contextual changes leads to the well-known perseveration effects of habits and overtraining.

## 2 Active Inference

We develop our argument within Active Inference: a framework that combines cybernetic ideas on the centrality of control and error-correction processes [1], [67], [83] with an inferential (Bayesian) scheme [27], [60], [73]. This section shortly summarizes the key aspects of Active Inference that are essential to understand the simulations reported in this article; a more detailed, formal introduction is provided in Appendix A.

Active Inference is a corollary of the free energy principle that casts decision-making and behaviour as a minimisation of variational free energy (or equivalently, a maximisation of model evidence or marginal likelihood). This means that perception and action (or policy) selection are treated as inference problems [2], [5], [16], [25], [42], [46], [55], [58], [70], [76], [77], [81]. Action selection implies evaluating the quality of a policy (or action sequence) *π* for each possible state an agent could be in – which corresponds to calculating the (negative) expected free energy of *π*, or **G**_*π*_. Importantly, policies are evaluated in relation to both their pragmatic or economic value (e.g., how well they achieve goals) and their epistemic value (e.g., how well they reduce uncertainty). To understand how pragmatic value is calculated, it is important to note that active inference absorbs goals into expected free energy in the form of prior beliefs about outcomes (that can be produced by acting). One can then formulate optimal behaviour as minimising surprise in relation to these prior beliefs. Furthermore, as minimizing expected surprise corresponds to minimising entropy, any policy that minimises expected free energy is, effectively, resolving uncertainty. The imperative of resolving epistemic uncertainty is thus part and parcel of free energy minimisation and it dissolves the exploration-exploitation dilemma; typically causing agents to forage for information until they are sufficiently confident to pursue their goals or prior preferences [26].

Active Inference is thus a quintessentially model-based scheme, which selects actions based on the prospective evaluation of the pragmatic and epistemic values of candidate policies. This future-oriented (or planned) behavior rests on the notion of the expected free energy attained following competing policies. More specifically, when an agent is in a particular (hidden) state, it can evaluate the quality of its policies in terms of the free energy attained under each policy:

$$G_{\pi }=\sum_{\tau =t}^{T}G\left(\pi ,\tau \right),$$

where

(1) $$G\left(\pi ,\tau \right)=-H\left[P\left(o^{\left(\tau \right)}|s^{(\tau }\right)\right)]\cdot {\hat{s}}_{\pi }^{\left(\tau \right)}-\left(\ln{\hat{o}}_{\pi }^{\left(\tau \right)}-\ln P\left(o^{\left(\tau \right)}\right)\right)\cdot {\hat{o}}_{\pi }^{\left(\tau \right)}$$

Here, *H* denotes entropy, and *P*(*o*^(*τ*)^|*s*^(*τ*)^) is the likelihood of the generative model, *P*(*o*^(*τ*)^) represents prior beliefs about future outcomes *o*^(*τ*)^ according the generative model and ${\hat{s}}_{\pi }^{\left(\tau \right)}$ and ${\hat{o}}_{\pi }^{\left(\tau \right)}$ are the expected states and outcomes under each policy at time *τ*, respectively. Heuristically, the first term represents the expected resolution (an expected value, mathematically) of uncertainty or ambiguity about outcomes, given hidden states, under the predictive posteriors over those states *Q*(*s*^(*τ*)^|*π*), while the second term expresses the divergence between the predictive posteriors and priors over outcomes *Q*(*o*^(*τ*)^|*π*) and *P*(*o*^(*τ*)^) (a derivation of Eq. (1) can be found in [26]). Intuitively, this scores the difference between predicted and preferred outcomes in the future, under the policy in question. This term is formally identical to the objective function of KL (Kullback-Leibler) control [36] and corresponds to expected risk in economics [85]. A softmax function of expected free energy under each policy **G**_*π*_ provides posterior beliefs about the best policy, from which the subsequent action is sampled.

The quality of policies (i.e., expected free energy) can be decomposed in several ways: in the absence of any ambiguity about the outcomes in any particular state, the expected free energy corresponds to the KL divergence between the predicted and preferred states. Preferred states are specified in terms of prior preferences. This means that Active Inference corresponds to risk sensitive control [33] when there is no ambiguity about outcomes (when there is ambiguity, the expected free energy also includes an additional epistemic component, please see [26], [53]). This scheme has been used to model waiting games [28], [29] the urn task and evidence accumulation [19], trust games from behavioural economics [45], addictive behaviour [72], two-step maze tasks [26], [53] and engineering benchmarks such as the mountain car problem [22]. It has also been used in the setting of computational fMRI [71].

In this article, we introduce a fundamental simplification of this Active Inference scheme, by showing how the evaluation of policies – and subsequent selection – can be finessed through *caching*. The rationale of this idea is that, if the contingencies mediating state transitions – and prior preferences – of an agent do not change, the quality of a policy from any given state will not change from trial to trial. This means the only things that change are the beliefs about the initial and subsequent hidden states the agent finds itself in. Thus, it is not necessary to re-compute the expected free energy **G**_*π*_ of policies on subsequent trials, because the only thing that changes are beliefs or expectations about hidden states. The latter can be accumulated from trial to trial so that the agent becomes more confident about the state it starts from.

Below we introduce three novel computational schemes that use caching within Active Inference and compare them during a simulated foraging task (the pseudocode of the three schemes is reported in Appendix B). Our simulations will show that (1) cached policy probabilities can be used to skip some or all the computations that underwrite Active Inference, thus entailing a more efficient mode of control (in terms of, for example, computational time); (2) these approximations are only valid in some circumstances, while in other cases they fail – producing characteristically inflexible behaviour and the perseverative effects of habits.

## 3 A simulated foraging task

**Fig. 1:**
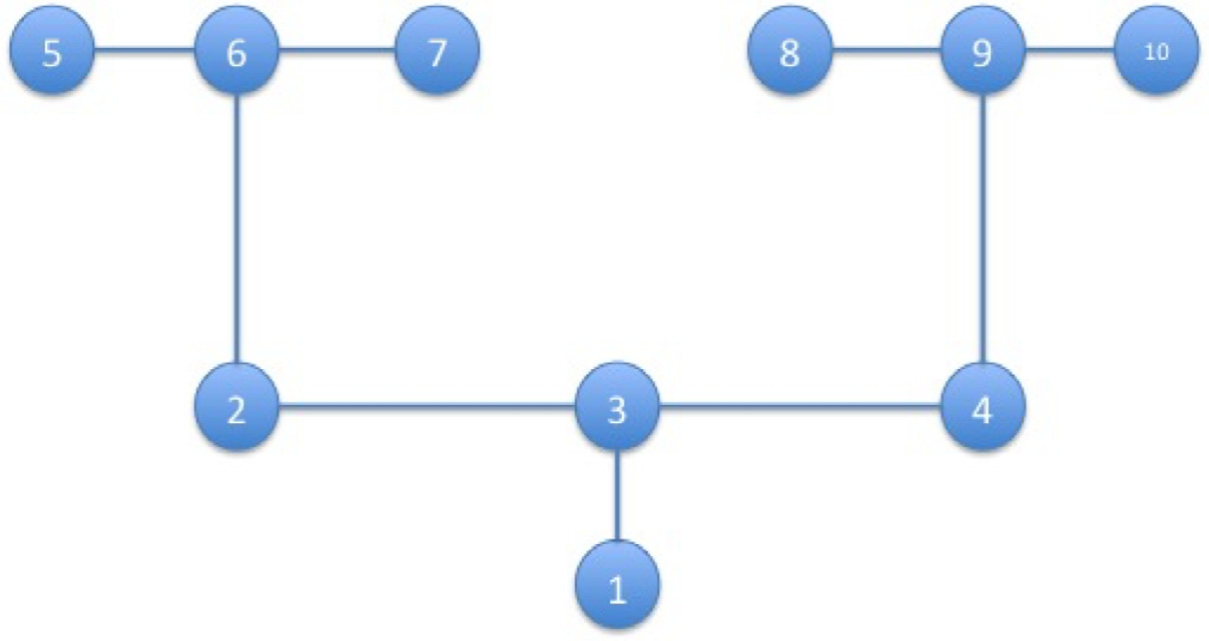
*The double T-maze scenario used in our simulations.* The agent always starts from the start location (location 1) and can collect rewards (only) at states 5, 7, 8 or 9 depending on the current context (A-D).

We illustrate how the caching mechanisms work by focusing on a simulated foraging task: a double T-maze with 10 locations. In this set-up, an agent (an artificial rat) starts from the initial location (location 1), and has to reach one of four reward locations (5, 7, 8, 10), see Fig. 1. At every trial, only one of the four reward locations is actually baited with a reward. The actual reward location depends on the current choice context, which can be conceptualized as reward contingency: context is A if the reward location is 5, is B if the reward location is 7; is C if the reward location is 8; and is D if the reward location is 10. The context is initially unknown to the agent – but since the agent is tested in the double T-maze for a number of successive trials (here, 40) it has the opportunity to learn which context it is in. Learning occurs because when the agent reaches (or not) a reward at the end of trial, it can (probabilistically) update its estimate of the current context (e.g., getting a reward in location 5 increases the belief to be in context A. Furthermore, the agent can transfer its context estimation to the next trial (i.e., it has prior knowledge that context is stable across trials with probability 99%) and thus, even if reward delivery is stochastic (here, reward are delivered in the “correct” location 95% of the times), it has the opportunity to accumulate information about its context across trials – or even infer the context has changed.

### 3.1 A schematic illustration of Active Inference

**Fig. 2:**
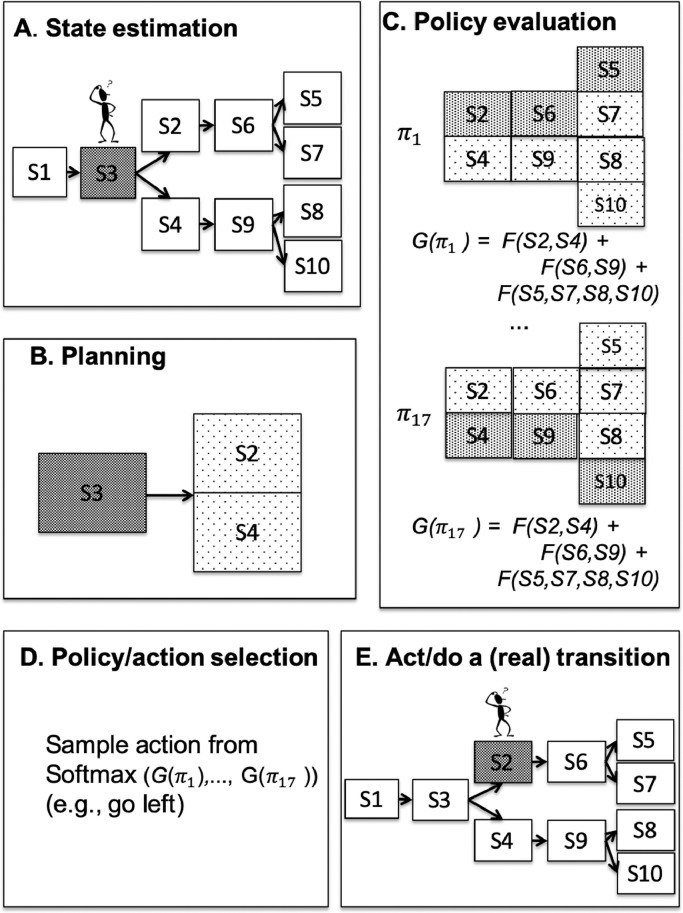
*Schematic illustration of the full active inference procedure.* This procedure comprises: (A) state estimation after the agent has collected an outcome in its current state (grey denotes high probability), (B) planning 1-step forward; (C) policy evaluation, *n*-steps forward, until the end of the policy; (D) policy / action selection; and (E) action. All these steps are repeated, until the end of the trial (in our simulations, 4 times, as the trial entails making 4 successive choices). Grey scale denotes probability distributions. Note that for simplicity, the generative model illustrated in this figure comprises 10 states (10 locations  ×  1 context), not 40 states (10 locations  ×  4 contexts) as in the simulations reported below. See the main text for details.

The simulated foraging task can be solved using the deliberative scheme of active inference, which uses a generative model to calculate the expected free energy **G**_*π*_ of (i.e., the path integral of the free energy expected under — see Appendix A.1 for a detailed description) the allowable policies, and then selects the next action using a (precision-weighted) softmax function of **G**_*π*_. A schematic illustration of the active inference procedure is sketched in Fig. 2 and a pseudocode is shown in Appendix B.

First, in stage A (Fig. 2A), the agent estimates its current state using its current observations and prior beliefs (not shown); the grey color of S3 denotes a high probability of being in that location. Second, in stage B (Fig. 2B), the agent does a one-step “forward planning” to predict the possible future locations (S2 and S4; light grey denotes a smaller probability compared to S3). Third, in stage C (Fig. 2C), the agent evaluates the quality of all its policies, i.e., their expected free energy (**G**_*π*_, see Eq. (1)), by considering the integral of the free energy along the states that compose the path defined by the policy. Note that if the agent’s generative (action-state) model is deterministic, as in this simulation, the path will comprise just a sequence of states. However, the model can be stochastic and thus each policy would entail a probability distribution of future states, as shown in Fig. 2 – and it is this distribution that will be evaluated. Fourth, in stage D (Fig. 2D), the agent transforms the list of **G**_*π*_ values of the policies into a probability distribution (using a precision-weighted softmax function) and then it samples an action from it. This means that it uses the expected free energy or **G**_*π*_ values of the different policies as (prior) probabilities for action selection. Finally, in stage E (Fig. 2E), the agent executes the action to induce a state transition in the “real world”. After this transition, the agent samples a new observation and starts again from stage A, and so on, until it reaches a termination (absorbing) state at the end of the maze.

As shown in previous simulations [26], [53] the Active Inference scheme leads to optimal (free-energy minimizing) behaviour: as the agent accumulates information across trials about its current context, it selects the reward location more frequently. However, this scheme is computationally costly and it requires engaging the full generative model for planning and policy evaluation that each trial. Our experiments below show that – in some circumstances – one can eschew parts of the deliberative processing by caching the probability distribution of the “values” of policies (i.e., their expected free energy **G**_*π*_), calculated during previous trials. In other words, there are cases in which using cached policy probabilities is more cost-effective that calculating them anew. This simplification is not just a nuance but can be seen as part of the free energy minimization imperative, if one assumes that agents believe they will avoid costly computations, analogous to model selection and the “simplification” of generative models by removing excessive parameters [18], [25], [60].

Below we introduce three approximate Active Inference schemes, in which the agent caches (probabilities of) **G**_*π*_ values of its policies, for each state. In the first scheme, the cached values are used to select the “best” policy, thus skipping entirely the computations of policy selection, planning and policy estimation. In the second scheme, the cached values are used for planning, whereas in the third scheme, the cached values are used for policy evaluation. In the next section, we introduce each scheme in detail, discuss the differences between them, and highlight similarities with caching mechanisms in biological and computational theories of (reinforcement) learning.

## 4 Results

We simulated the behaviour of an Active Inference agent in the double T-maze shown in Fig. 1 for 40 trials (all results are an average of 100 simulations). The agent starts always from location 1 in the maze. The initial hidden context is A (i.e., reward location is 5) from trial 1 to trial 20, and then it becomes context D (reward location is 10) from trial 21 to trial 40 – thus requiring the agent to change its policy.

The generative model used for the simulations is shown in Fig. 2. It comprises 40 states (10 locations  ×  4 contexts); 20 observations (10 locations  ×  2 cues: red = reward, white = no reward); 17 policies, which cover exhaustively the possible action sequences in the maze. Note that only 4 of these policies potentially lead to a reward, i.e., (up, left, up, left), (up, left, up, right), (up, right, up, left), (up, right, up, left).

### 4.1 Scheme 1: using the cache during action selection

Suppose an agent has to solve the foraging task shown in Fig. 1 many times. If the agent remembers or caches the “value” or “quality” of its policies from previous trials, then all it has to do is to take (at the beginning of stage A) the maximum of this value under its beliefs about current hidden states; for example, that it is starting from location 1 in context A. This avoids having to reassess the B,C and D stages of the procedure, and thus to re-compute the policies and engenders habitual behaviour. Clearly, this will only work if we assume one of the alternative policies constitutes a viable solution for the current task. However, under this assumption, the resulting scheme provides a graceful connection between goal-directed active inference and habitual behaviour; the habit is selected automatically when the agent is sufficiently confident that it will result in preferred outcomes. The nice thing about this scheme, is that habit selection is very simple: if, at the beginning of each trial (before stage A), the maximum value of the expected distribution, with respect to the expectations of the final state, over the policy beliefs conditioned by the hidden states, namely:

(2) $$E_{{\hat{s}}^{\left(T\right)}}\left[P\left(\pi |s\right)\right]=\sum_{i}{\hat{s}}_{i}^{\left(T\right)}P\left(\pi |s_{i}\right)$$

is higher than a given reference value – for example, $p_{\mathrm{th}}=0.90$ – or equivalently, if there is a policy whose probability is higher than 0.9 – then it can be selected automatically; otherwise, the quality of all policies can be re-evaluated, to ensure that prior preferences or task contingencies have not changed.

In contrast to standard Active Inference, here the agent caches the probability of all policies in each state *P*(*π*|*s*) (i.e., the precision-weighted softmax of the expected free energy **G**_*π*_ of policies), for each state. At the beginning of each trial, the agent does not use Active Inference; rather, it firstly checks if the cache of the last context it experienced (in the previous trial) includes a policy whose probability is higher than *p*_th_. If this is the case, it selects (deterministically) this policy. If not, it adopts the usual active inference scheme shown in Fig. 2 and updates the cache: the policy probability values *P*(*π*|*s*) are updated using a sequential update (delta) rule:

(3) $$P{\left(\pi |s\right)}_{t+1}=P{\left(\pi |s\right)}_{t}+w_{\hat{s}}^{\left(t\right)}\left(\hat{\pi }-P{\left(\pi |s\right)}_{t}\right)$$

In Eq. (3), the updated policy probability (at time t+1) considers the cached policy probability (at current time) and a prediction error term, which compares expectations about policy probabilities and their cached probabilities (at time t−1). The prediction error term is weighted by $w_{\hat{s}}^{\left(t\right)},$ which corresponds to the probabilities of each policies to take the agent occupying the expected state $\hat{s}$ at the current time *t*. This implies that, the more confident the agent is about being in a particular state, the more it will change policy expectations, conditioned on that state. This updating procedure is used for all policies in all the states that the agent entertains.

**Fig. 3:**
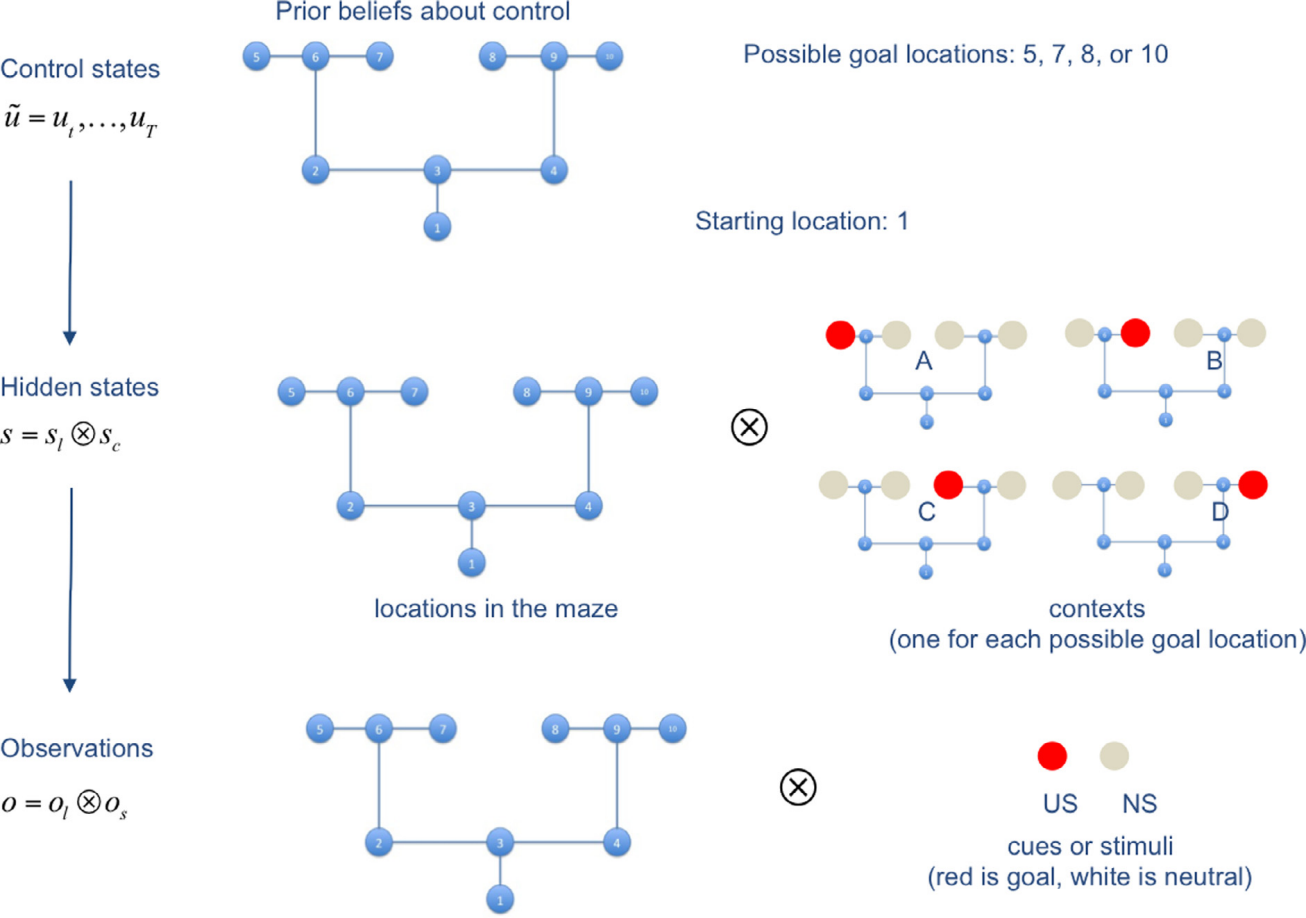
*Schematic illustration of the generative model.* The generative model comprises 10 control states, 40 hidden states and 20 observations. Red is a reward observation, while white is a neutral, non-rewarding stimulus. This figure shows that the starting location is always 1, but it can correspond to 4 different states depending on the context (e.g., state 1 if the context is A, state 4 if the context is D). There are 4 potential goal locations: location 5 if the context is A (which corresponds to state 17), location 7 if the context is B, location 8 if the context is C, and location 10 if the context is D (which corresponds to state 17). Note that in our simulations, we only use the two contexts A and D. We constructed 17 policies (not shown here) that cover the possible moves of the agent. The two most important policies are policy 1 = up, left, up, left, which is the best policy under context A, and policy 2 = up, right, up, right, which is the best policy under context D.

**Fig. 4:**
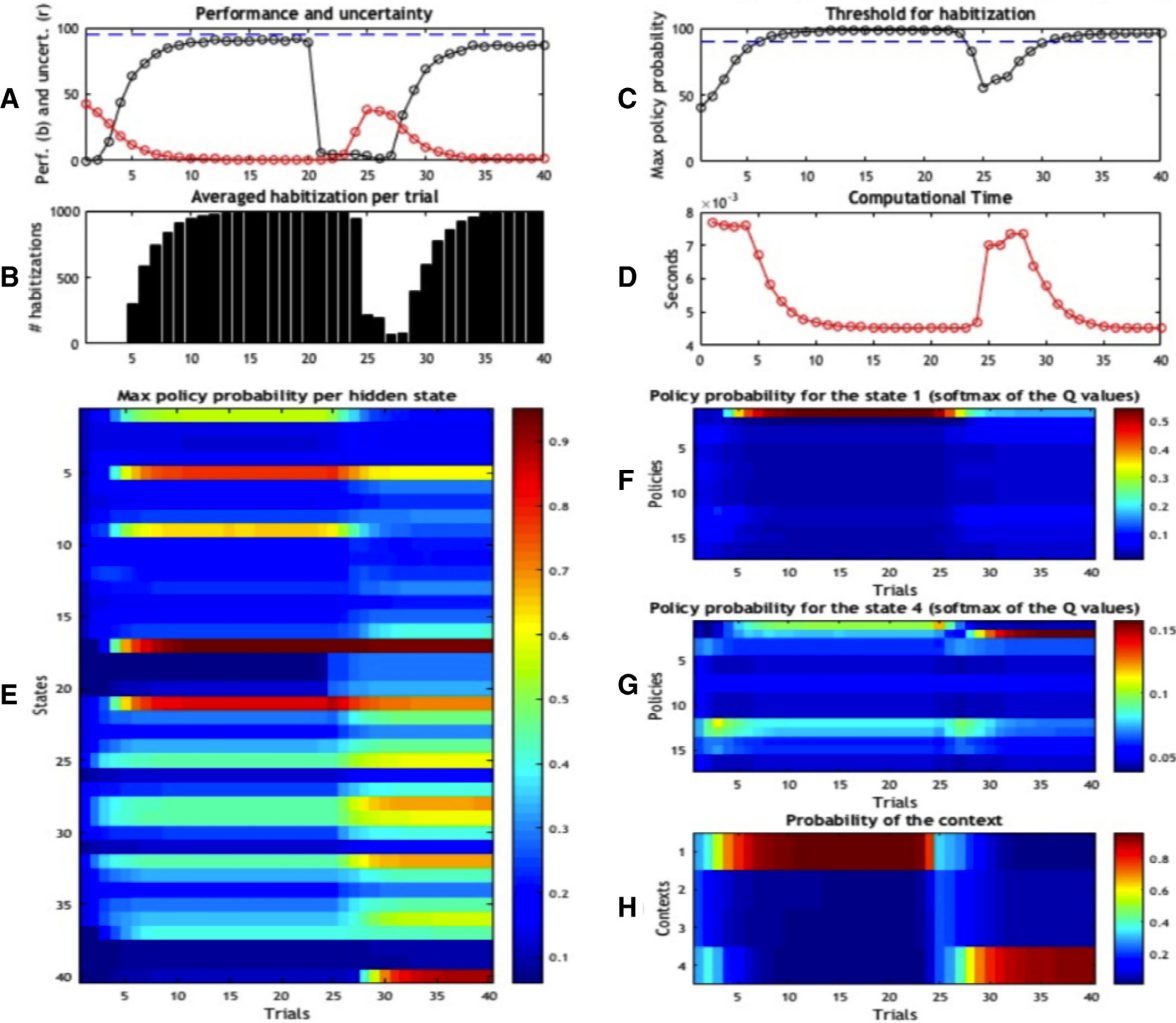
*Simulation results of Scheme 1.* Agent’s performance (A–C) and beliefs (E–H) during the 40 trials. All results are the average of 100 repetitions. (A) Performance (black) and uncertainty (red) of the agent in the 40 trials. Note that we changed the context (and reward contingencies) at trial 21. (B) Average percentage of habitised behaviour. (C) Probability of the best policy; the threshold for habitisation is 90%. (D) Computational time required by the scheme. (E) Maximum probability of a policy, for every state. Note that states are calculated as “locations  ×  contexts”; thus, for example, states 1, 9, 5, 21 and 17 form the correct path for context A, and correspond to locations 1, 3, 2, 6 and 5 of context A in Fig. 1. (F) Probability of the 17 policies when the agent is in state 1; i.e., the starting location in context A. (G) Probability of the 17 policies when the agent is in state 4; i.e., the starting location in context D. (H) Contextual estimate; i.e., probability that the right context is 1 (A) to 4 (D).

The functioning of the first caching scheme is illustrated in Fig. 4. The first four panels (Fig. 3 3A-D) show the results of the foraging simulations, averaged across 100 replications. Panel A represents the performance of the agent over trials 1–40 (in black) and its uncertainty about the current context (in red). After the first few trials, accuracy increases and uncertainty decreases. This ramping of accuracy (and decrease in uncertainty) is due to the fact that rewards are collected probabilistically (rewards appear 95% of the time in the contextually correct location; i.e., location 5 in context A) and actions are selected stochastically during active inference. However, the context changes at the 21th trial. Because the agent is not immediately aware of the contextual change, it will initially select the old, wrong policy – and fail. However, after a few trials, its performance starts to recover, achieving a high level of accuracy after few trials. Panel B reports how many times (averaged across 1000 repetitions) choice was habitised, for each trial. This panel shows a progressive transfer of control from deliberative choice to automatic habit selection, when uncertainty falls significantly (around trial 5), then a second transfer of control back to deliberative choice (shortly after the contextual change of trial 21), and a third transfer of control when uncertainty is resolved again (around trial 30). Panel C shows the same phenomenon, but from another angle: it reports the (average) probability of the highest policy, and shows that (on average) the best policy surpasses the habitisation threshold (here, $p_{\mathrm{th}}=0.9$) around trial 5, then falls below the threshold shortly after the contextual change of trial 21, and surpasses the threshold again around trial 30.

These results show that despite habitisation, context sensitivity is preserved. This is because policy selection is based upon averaging the quality of policies over beliefs about hidden states – that include contextual factors. This means that if the agent encounters a change in context, the outcomes will induce a loss of confidence about the context it is currently operating in. This will reduce the relative probability of the habit, enabling a new policy to be evaluated online (and eventually the formation of another habit). In other words, the fact that policies are context-specific allows the agent to recover from wrong habits, after it notices a contextual change (thus, with some delay).

What are the benefits of selecting policies automatically rather than using the full deliberative scheme of active inference? Panel D illustrates the benefits of caching **G**_*π*_ values in terms of a measure of complexity: the (computational) time of the scheme. Initially, computational time decreases towards a plateau that corresponds to the habitisation stage. Computational time rises again following the contextual switch and then decreases smoothly until the last trial. On average, the time for the execution of a single trial is 0.0054 s. Note that computational time is just one of several ways to characterize the “costs” of the different solutions; it is used here as a proxy for various kinds of resources (e.g., attention, memory and planning) that need to be allocated to deliberative control, see [74].

The agent’s internal states that lead to habitisation and transfer of control are illustrated in Fig. 4E, which shows the probability of the best policy for each of the 40 states, over time. Although this figure shows values for each state (location  ×  context), in our simulations we only use the maximum value for each context, which corresponds to state 17 (in context A) and state 40 (in context D). In other words, the agent would only need to know (cache) the value of its best policy in each context (4 items rather than 40). However, showing all 40 states in Fig. 4E illustrates the progressive development of a graded representation of the value of (the best policy in) states, which has analogies with the notion of a value function in reinforcement learning (although it is used differently in this simulation), see [78]. One can see that this probability increases over the first 20 trials for all the states along the correct path under context A (i.e., states 1, 9, 5, 21 and 17, which correspond to locations 1, 3, 2, 6 and 5 under context A). Note that the probability is already high at state 1, which corresponds to the start location, but increases with successive moves towards the correct goal site (this is expected: in active inference, policy values can only increase along the path if the agent collects no new information).

After the contextual change at trial 21, the probabilities of the states along the correct path under context D start to increase. At the same time, the probabilities of the states along the correct path under context A begin to decrease (although they remain significantly higher than the probabilities of other paths). This is because, after the changing context, the agent becomes uncertain about the current context (see Fig. 4H); hence it updates the cached policy expectations under context A fallaciously. In other words, when it recalculates policy expectations, it updates policy expectations for all contexts A–D (albeit with a rather low weight w), rather than only in the states of context D, in which it currently occupies. Still, because the agent identifies the contextual change within a few trials, it quickly begins to update only the states of context D and does not completely “wash out” the cache for context A. In other words, after a few trials, the agent correctly identifies the existence of two different contexts (A vs. D), each affording a different policy. Context sensitivity is important because it precludes catastrophic forgetting – the replacement of old (but useful) memories when context changes, at the expense of a larger state space.

Panels F and G show the probability of the 17 possible policies at the starting location in context A (i.e., state 1) and context D (i.e., state 4), respectively^1^. This probability is calculated by simply re-normalizing the **G**_*π*_ values of the different policies in states 1 and 4 of Panel D. As shown in Panel F, the probability of the best policy (policy 1 = (up, left, up, left)) in state 1 increases over time during the first 20 trials, in which the context remains stable. In other words, when the context does not change, policy selection becomes essentially deterministic – because the agent has identified the correct policy and, consequently, there is no need for exploration or epistemic foraging [26]. In these conditions, caching **G**_*π*_ values is clearly more parsimonious than re-computing them. As shown in Panel G, in correspondence of the contextual shift at trial 21, the agent has no policy that is fit for purpose for the new context. This is because it has not identified the contextual change yet (i.e., it still believes it is in context A, see Panel H) and keeps selecting the best policy of context A (policy 1) for a while. This perseveration is a typical effect of habitual behaviour and persists until the agent correctly infers the changing context. When the agent starts experiencing surprising outcomes (no reward) for a few trials and becomes thus uncertain about the current context, it starts exploring the task contingencies, as in the initial experimental trials, until it identifies a good policy (policy 2 = up, right, up, right) for the new context D. At this point, the probability of this new policy increases and behaviour can be habitised again.

Panel H shows that after the contingency change at trial 21, the agent incorrectly believes it is still in context A. This is because, in our simulations, outcomes are collected probabilistically (95% of the times); failing to solicit an expected outcome lowers the confidence about the context but does not necessarily mean that a change of context has occurred. Furthermore, the agent has a prior belief that context will remain stable across trials 90% of the times (which enables it to use its knowledge accumulated during past trials at the beginning of a new trial). As a consequence, changing beliefs about context thus requires a few trials. During these trials, the agent needs to explore other paths / policies, in order to resolve uncertainty (i.e. expected free energy) about the new context is B, C or D (and to find a good policy for the new context).

In summary, this caching scheme solves the foraging task efficiently and is more efficient than the full active inference scheme. However, this scheme only considers whether a good policy was available in the last context (during the previous trial) and selects an action without estimating the context it finds itself in at the beginning of a new trial. This is not a problem (except during contextual changes) in our simulations, where the context remains stable for 20 trials. However, in other situations – such as when the context is more volatile (e.g., A-D-A-D) – this scheme would perseverates with the wrong policy – even if cued about the new context. This limitation is addressed by the next caching scheme, which infers the context before selecting a (deliberative or habitual) policy.

### 4.2 Scheme 2: using the cache during planning

The second caching scheme we considered uses the cache during the planning phase (stage B, illustrated in Fig. 2B) of Active Inference. If, during planning, the agent expects to be in a state having a cached policy probability $E_{{\hat{s}}^{\left(t\right)}}\left[P\left(\pi |s\right)\right]$ exceeds the threshold $p_{\mathrm{th}}=0.9,$ it selects the policy, thereby skipping the rest of Active Inference evaluation. Otherwise, if the probabilities of all the expected policies are less than the threshold, it re-computes the policy **G**_*π*_ values (and probabilities), updates the cache as in the first scheme, and proceeds with the usual Active Inference procedure. In contrast to the first caching scheme, here the agent infers the current context and starts the planning procedure before selecting (eventually) a habit. This makes this second scheme slightly more costly than the first, as the agent needs to infer its current state and form beliefs about the future hidden states expected under a policy, but still less costly than the full active inference procedure.

**Fig. 5:**
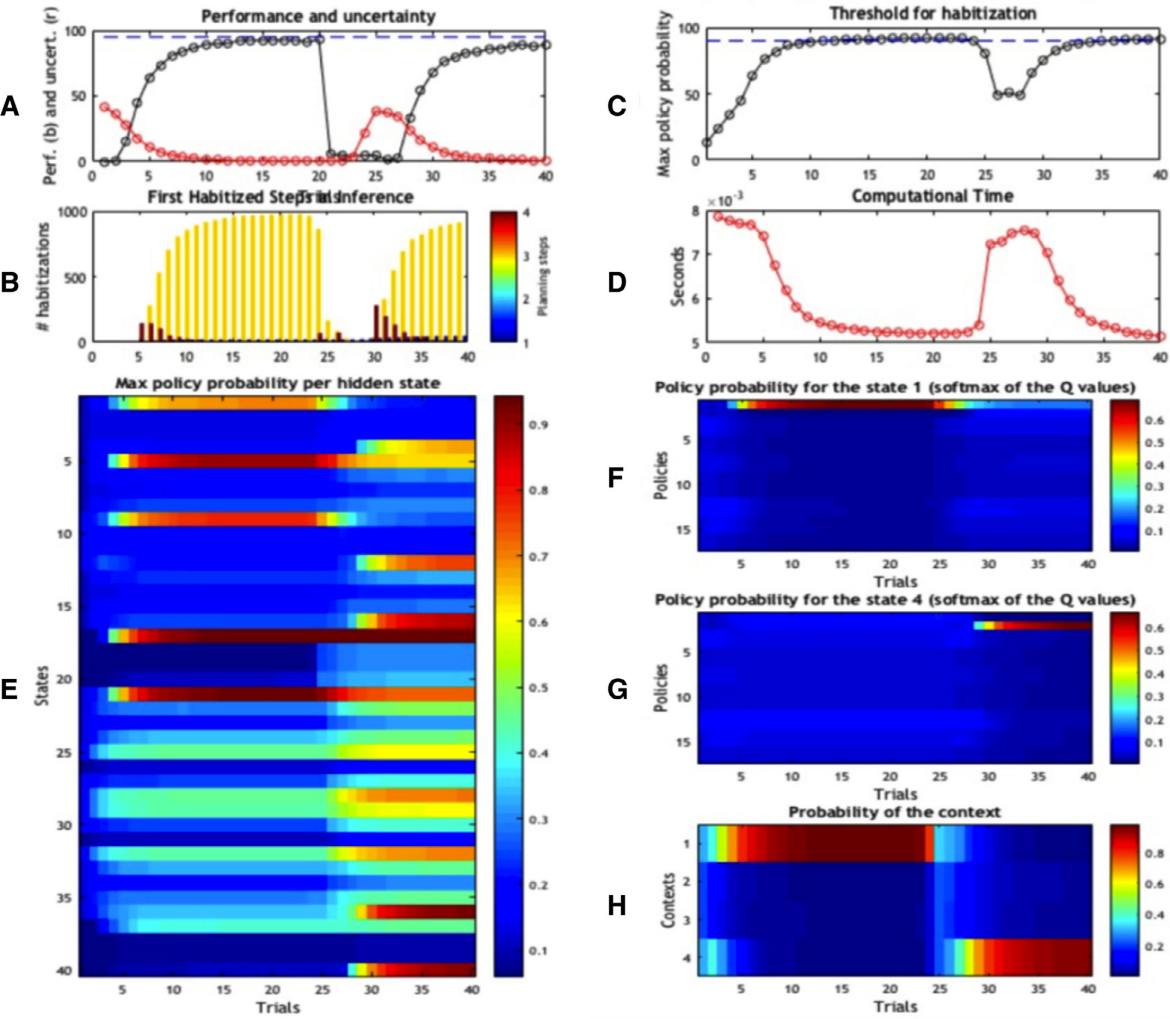
*Simulation results of Scheme 2.* Panels use the same format as in Fig. 4, except for Panel B, where results are colour-coded, reflecting the fact that behaviour can be habitised at the first (blue), second (azure), third (yellow) or fourth (red) decision point.

Fig. 5 shows the results of this simulation using the same format as Fig. 4. Panels A–D are very similar in Figs. 4 and 5, which implies that despite the different caching mechanisms, the performance of the two schemes is qualitatively similar. However, there are some important differences due to the particularities of the two schemes. The most important difference between this scheme and the previous scheme emerges when one considers that, in the current scheme, deliberative and habitual behaviour can coexist in the same trial; i.e., the agent can deliberate at the beginning of a trial and then complete the trial with a habit. In our simulations, completing each trial requires 4 choices. In contrast to Figs. 4B, 5B is colour-coded to illustrate when, during a trial (i.e., at the 1st, 2nd, 3rd or 4th choice) choice is habitised. The results show that choice is under deliberative control in the first trials, as in the first scheme. From the 5th trial, the agent begins to habitise the last part of the trial (i.e., the 4th choice) and successively earlier parts of the trial (i.e., the 3rd choice). This means that, over time, the agent becomes more confident about the best policy earlier in the trial – although with our choice of parameters (e.g., the 90% threshold) choice is never habitised from the beginning of the trial. In short, this scheme underwrites a progressive reduction of computational time, which is modestly longer, on average (0.006 *s*.) than the first scheme.

A further difference emerges if one considers that the current scheme is slightly slower in reaching the threshold for habitisation, both before and after a changing context: compare Figs. 4C and 5C. While the first scheme considered the context inferred on the last trial (e.g., state 17), the second scheme only considers the (agent’s belief about the) next states it can visit, which have on average lower policy values. It thus takes longer for this scheme to reach the threshold for habitisation. For the same reasons, policy probabilities are generally greater in this second scheme compared to the first (compare Figs. 4E and 5E); this is especially evident in the first states visited by the policy (e.g., state 4 after the contextual change), which are important in this second scheme but somewhat irrelevant in the first. Of course, it would be possible to compensate for the slight decrease of performance of the second scheme by setting a lower threshold for habitisation, or a “relative” threshold that compares the probability of the first and second-best policies (e.g., a policy would be selected if it is 90% more probable than the second-best).

### 4.3 Scheme 3: using the cache during policy evaluation

This third scheme is similar to the second, but uses cached values during policy evaluation (stage C, illustrated in Fig. 2C), not during planning. In Active Inference, evaluating the **G**_*π*_ value of a policy requires a path integral of the expected free energy under that policy (see A.1 for details). Using the third caching scheme, the agent terminates the path integral when it finds a policy for which $E_{{\hat{s}}^{\left(\tau \right)}}\left[P\left(\pi |s\right)\right],$ the expected value over ${\hat{s}}^{\left(\tau \right)}$ of the policy probability, is higher than 0.9, and selects this policy. Otherwise, it completes the path integration normally and updates the cache. This scheme has thus some analogies with algorithms in artificial intelligence that perform “moderate” forward search, until they find a reliable cached value or chunk [7], [30], [38], [59].

**Fig. 6:**
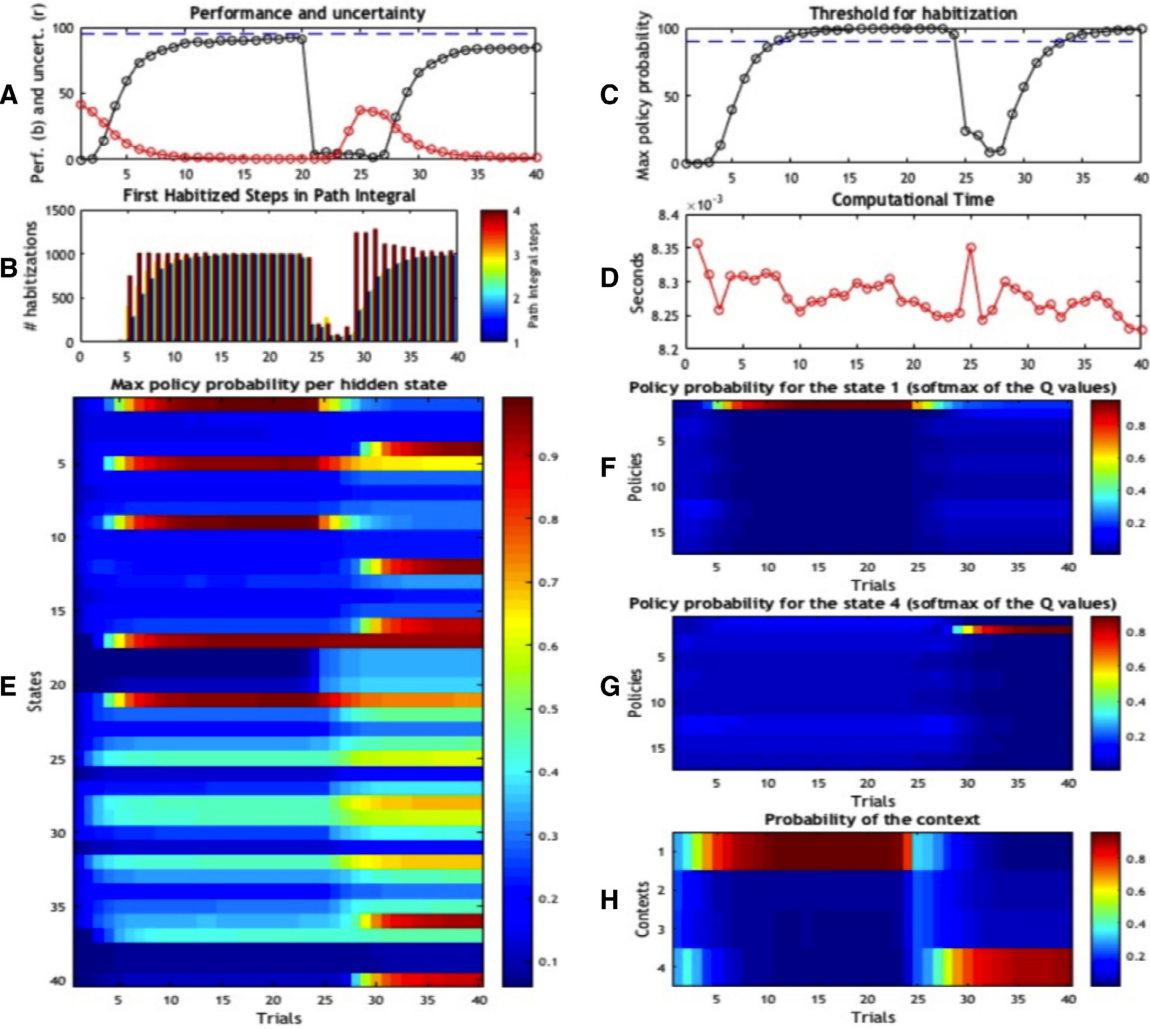
*Simulation results of Scheme 3.* Panels conform to the same format as in Fig. 4.

As shown in Figs. 6, accuracy (Panel A) and the probability of the best policy (Panel C) rise slightly more slowly in this scheme compared to the previous scheme. However, the values reached by the habitual policy (Panel C) are slightly higher than the second scheme, and comparable with the first. This is because the third scheme performs prospective predictions (during the policy evaluation) and, like the first scheme, can potentially tap the (higher) probability values cached under the terminal states (e.g., states 17 and 40). As in the second scheme, in the third scheme deliberative and habitual choice can coexist in the same trial. However, while in the second scheme, habits were most common for the last part of the trial (i.e., the 3rd and 4th choices), here they are equally common throughout the trial (i.e., the 1st, 2nd, 3rd and 4th choices). This difference can be seen by comparing Figs. 5B and 6B. The ability to habitise the initial parts of the trial depends, again, on the fact that this scheme performs prospective counterfactual predictions. It is also evident in Panel C that the probability of the best policy drops significantly after the contextual change in trial 21; reflecting the fact that the relative values of policies are low at that point. Finally, computational time is variable across trials, with a mean time execution for a single trial equal to 0.0083 *s*. Given that this scheme uses policy evaluation more often, it is slightly slower than the previous schemes.

## 5 Discussion

We have considered the possibility that an Active Inference agent caches (i.e., memorizes) the probability of policy (under the current state) from previous trials, in order to reduce the computational costs required to calculate them anew at each new trial. Using caching methods, either in isolation or in combination with search-based methods, is popular in various areas of reinforcement learning [78], [79], machine learning and artificial intelligence [30], especially when it is necessary to address large state spaces. However, caching per se is not without costs: human and animal experiments have shown that caching (and more generally reusing old solutions to address new problems) can come at the expense of (relative) behavioural inflexibility in the face of contextual changes, which is typically associated with habitisation and/or automatic action control [3], [47]. Animals and artificial control systems thus face the problem of trading-off the flexibility of deliberative strategies and the parsimony of habitual strategies, and need adaptive strategies to transfer control between them.

In the novel approximation of Active Inference illustrated here, the agent caches the probability of its policies, which are calculated as a function of their expected free energy **G**_*π*_ is state-specific (i.e., what is cached is a Softmax function of **G**_*π*_, for each state). Of note, the expected free energy **G**_*π*_ of policies used here is estimated on the fly using deliberative inference. Despite we heuristically refer to **G**_*π*_ as a “value” of policies, it is different from the notion of a value function (of actions or action-states) in model-free reinforcement learning, which is usually learned by trial and error [82].

To motivate the novel caching scheme, we have exploited the fact that the quality of policies, conditioned upon hidden states, is constant over trials; provided contingencies and prior preferences do not change. In brief, this means the only quantity that can change policy selection is the prior distribution over the initial state – where this prior is based upon the posterior beliefs from previous trials (e.g., one can infer a contextual change from the fact that it has failed to collect a reward in the usual location). Thus, an agent that caches the quality (or the probability) of policies can safely reuse these values, unless a contextual change occurs. This leads naturally to a theory of habit formation, in which habitual policies form by caching the computations of a deliberative controller [11], [12], [23], [60].

We have illustrated three possible uses of caching, each associated with a specific aspect of active inference: action selection, planning and policy evaluation. Although these are just examples of the many possible uses of a cache, they have analogies with theories that emphasize that habitual behaviour eschews inferential processes entirely (first scheme), that one can combine deliberation and habits even within a single task or a single trial (second scheme), and that one can perform a limited or “moderate” forward search until one finds a reliable cached value, see [7], [30], [38], [47].

The three caching schemes explored produce similar behavioral results; but yet there are some differences amongst them, which might reflect different kinds (or phases) of habitisation. The first scheme is slightly faster than the other two, as it uses the cached values to elude the inferential mechanisms of active inference – a “trick” that can however produce maladaptive behavior, if contexts are varied too often. In other words, this scheme would fail to update a policy at the beginning of a new trial, even if it is cued about a contextual change, producing inflexible perseveration. Consider the case of a person who uses the same coffee machine every morning. Even if one day he was told that the coffee machine is broken, he would fail to assimilate the contextual change into his policy selection and thus follow the usual habit (e.g., to try operating the coffee machine). The second and third schemes are less prone to this problem, because they consider the current context before selecting an action. However, unlike the full active inference, the second and third schemes do not update the value of policies based on all available knowledge, and might fail when some contingencies change more quickly than the update of the cache (e.g., if the coffee machine is moved, they might sometimes go to its previous location). Furthermore, given that these schemes implement a form of bounded search for (cached) policy probabilities, they may become stuck in local minima; for example, they might select a suboptimal policy that reaches a smaller but proximal reward, rather than an optimal policy that reaches a bigger but more distal reward. These problems are exacerbated by the fact that an agent using caching mechanisms does not explore properly. In active inference, the balance between exploration and exploitation depends on the relative importance of epistemic and pragmatic values, which are jointly considered during policy evaluation [26]. Using caching mechanisms prevents updating policy values in the correct way when epistemic value changes, thus leading to suboptimal exploration. All these phenomena – perseveration, insensibility to contingency changes, short-sightedness and suboptimal exploration – have been variously associated with habitual behaviour. The fact that they may be partially dissociated under the different caching schemes explored here suggests that one can use these methods to probe the existence of different kinds of habits in humans and other animals.

Most theories of habits emphasize reduced behavioural variability, too [84]. In keeping, the caching methods explored here reduce variability of behavior. However, in active inference, action selection tends to become deterministic over time, when uncertainty decreases, even without caching or the transfer of control to a habitual controller. This is due to the convergence of various factors. First, the **G**_*π*_ value computations become increasingly more similar over time, given that the context does not change (this is why it is possible to cache them). Second, policy precision increases with time, as uncertainty about the current context decreases. Policy precision plays the role of the temperature parameter in the softmax used for action selection; hence, the higher the precision, the more action selection becomes deterministic. Finally, epistemic value tends to disappear as uncertainty about the prevailing context decreases, further reducing the need for exploration [26], [53]. Hence, in Active Inference, reduced behavioural variability does not necessarily imply a habit or the transfer of control from deliberate to habitual mechanisms.

Another hallmark of habits is inflexibility in the face of contextual changes. The general idea is that, if there was a transfer of control from deliberative to habitual (or automatic) mechanisms, an agent should not readapt its behaviour to novel circumstances (e.g., a change in reward contingencies), and/or it would fail to transfer back control from habitual to deliberative mechanisms when necessary. Here, however, there is an important difference between the behavioural paradigms that are used in animal learning (e.g., devaluation after overtraining, see [3]), which show a complete inflexibility after overtraining, and the somewhat less extreme situations that everybody experiences; e.g., recall the driver example in the introduction, in which a contextual change or a possible danger is detected, albeit with some delay, even when one is under automatic control [47]. We argue that these two situations can be reconciled if one considers the important role of context monitoring (Panels H of Fig. 4, Fig. 5, Fig. 6). In our simulations, the agent monitors the current context even when it uses cached policy values. Shortly after a contextual change, it perseverates with the wrong behaviour, because it fails to notice the contextual change (this occurred in our simulations in trials 21–24). However, after that it can transfer control back to deliberative mechanisms [47]. Notice that the agent is relatively impaired for a few trials after a contextual change, because it has to estimate the next context and find a good policy to deal with it – by exploring under the control of deliberative mechanisms. However, the agent does not show the strong form of inflexibility that is sometimes reported in the animal learning literature [3], with a long-lasting perseveration, even when contextual changes are cued. The second and third approximate active inference schemes illustrated above are especially resistant to excessive forms of behavioral inflexibility, as they explicitly consider their current context before action selection and can thus more easily monitor contextual changes.

One might speculate that the strongest forms of inflexibility demonstrated in the animal learning literature (for example, after overtraining) depend on a failure of monitoring contextual changes, which precludes transfer from habitual to deliberative control. Indeed, overtraining creates the preconditions for reduced contextual monitoring (in addition to habit formation), by biasing towards an underestimate of the volatility (or rate of change) of the environmental contingencies. This follows because, if the animal always operates in the same environment, its prior belief that the context will change may become extremely low – and it thus may fail to infer (or attend to cues) that the context has changed; or to update appropriately its contextual estimation based on novel evidence [26], [51]. This hypothesis may explain why overtrained animals become insensitive to reinforcer devaluation and other procedures that assess the balance between habitual and deliberate choice [3], [4], [9], [31], [32].

From the perspective of active inference, this sort of context insensitivity emerges as a consequence of treating policy selection as Bayesian model selection. In other words, by associating policies with models of ‘how to behave’ one can articulate a failure to consider certain models (e.g., a goal directed policy) in terms of Ockham’s window. Ockham’s window provides a range of prior probabilities that identify a set of models from which one is selected. If the habitual policy is, a priori, sufficiently more likely than any other policy, it effectively precludes alternative policies from consideration. Future experiments that distinguish policy selection from context monitoring may help test these and other ideas.

It is worth noting that the forms of habit selection explored in this paper will only work when the following conditions hold: (1) the probability transitions under each policy are known; (2) the prior preferences are fixed; (3) each state is visited once and only once; (4) one of the policies entertained by the agent is a potential habit. These conditions constitute the requirements for a state-action policy. In other words, if there is a unique ‘best’ policy from any (initial) state, then it is possible to identify and select this policy given precise beliefs about the initial state (and cashed **G**_*π*_ or probability values). In a previous work [23], we considered a related problem, in which the optimal habit is not included in the repertoire of policies – but can be learned using sequential policy optimisation (i.e., goal directed active inference via estimating **G**_*π*_ values at each time point). This allows de novo habits to be learned under the hierarchical supervision or contextualisation of goal directed active inference (with sequential policy optimisation). In other words, agents can learn habits through minimising their expected free energy – and then select them from an augmented repertoire of policies, in a way that resembles the first scheme presented above (i.e., skipping planning and policy evaluation procedures). Both the caching method presented here and the policy learning method presented in [23] aim at exploring the various ways humans and other animals can implement intentional actions, while (under certain conditions) alleviating the computational burden of deliberative processing.

It is possible to merge these approximate active inference schemes or design new ones that enhance the agent’s performance. For example, it would be easy to design caching mechanisms that combine elements of the first and the second scheme, in which (1) only one policy is cached for each context, with a probability that – as in the first scheme – reflects the value of the policy at the last state experienced under each context (e.g., state 17 for context A and state 40 for context D), (2) the agent estimates its current state / context, as in the second scheme. This scheme would give behavioural results that are similar to the first policy (results not shown), but will be less prone to rapid contextual changes after learning the most appropriate policy in each context. In the setting of this article, we were not interested in testing all the possible caching schemes or variants, but in demonstrating the utility of caching in the transfer control from deliberate to habitual mechanisms, and vice versa, as a function of confidence in the current context and policy – and to understand when and why behaviour becomes inflexible.

These problems have been widely addressed in other domains such as biological and computational (reinforcement) learning. Below we illustrate the main similarities and differences between our proposal and related schemes in reinforcement learning.

### 5.1 Relations with previous theories of goals and habits

Our proposal differs in many respects from widespread conceptualizations of goals and habits in brain and behavior [15]. A common assumption in the literature is that deliberative and habitual strategies of choice may correspond to two different control schemes that operate in parallel and compete in the brain. In particular, the *multicontroller hypothesis* proposes that control can be flexibly allocated to one of two controllers, one for deliberative and one for habitual choice, based on their relative uncertainty [10]. These two controllers map to model-based and model-free methods of reinforcement learning, respectively. While the former (model-based) method entails a form of prospective evaluation of future rewards, the latter (model-free) method uses cached action values for action selection. The multicontroller framework thus explains the transfer of control from deliberative to habitual choice on the basis of the fact that, in general, the model-based controller has lower uncertainty in early trials, and the model-free controller in later trials [15]. Recent developments of this heuristic have shown that – from both a computational and a biological perspective – the two controllers might be combined and realize a continuum [40], [48], [59] or a hierarchy [12], [60], rather than being strictly separated or alternative. Furthermore, it has been suggested that model-based and model-free controllers might operate sequentially rather than in parallel, and it is only when model-free mechanisms are insufficient that model-based computations are used to complement them – thus realizing a mixed controller [62]. Finally, it has been argued that the arbitration between deliberative and habitual modes might not depend on uncertainty only, but on more complex forms of cost-benefits computations [37], [59].

Our proposal shares some similarities with the multicontroller hypothesis (and related views), such as the idea of mapping deliberative and habitual mechanisms into distinct computational processes; and the fact that environmental uncertainty is one of the factors determining the transfer of control from deliberative to habitual strategies, and vice versa. However, our proposal differs from the multicontroller hypothesis, in many respects.

In the framework described here, deliberative processing maps to active inference, whereas habitual processing results from caching deliberative policies – or, when they are not sufficient, learning habitual policies on top of previous deliberative choices [23]. This view has a number of implications. First, the distinction between deliberative and habitual schemes maps to a mechanistic distinction between belief-based (active inference) and belief-free schemes. In the current implementation, which uses cached policy probabilities, the latter (belief-free) scheme is essentially stimulus-response, and is thus value-free; i.e., it does not explicitly include state value representations [23], [44], [60]. This is markedly different from the idea that habitual mechanisms correspond to model-free RL, which uses cached action values [10]. The perspective advanced here is thus more compatible with recent theories that cast doubt on the fact that value representations are central to habits and propose that habits encode stimulus-response pairs instead; see [43], [44], [84] for a recent review.

Furthermore, our schemes suggest that the transfer of control from deliberative to habitual processing may depend on the increased reliability of cached policies, when contingencies do not change, whereas the transfer of control from habitual to deliberative processing is not immediate but calls on a contextual estimation. Note that in these schemes, there is a unique mechanism (or threshold) that determines the passage from deliberation to habits, and vice versa. This is because the cached policy probabilities that generate habits are associated with specific states or contexts. If the agent is in a state for which a cached policy probability exceeds the threshold, it can select it direcly – thus instantiating a transition from deliberative to habitual choice. However, if the agent detects a contextual change to a state for which none of its policy probabilities exceed the threshold, then it has to use full active inference – thus determining a transition from habitual to deliberative choice. The same transition can occur when the agent becomes unsure about what state it is in. This points to the recognised link between environmental uncertainty and deliberative behaviour. In turn, this implies that if an agent under habitual control keeps monitoring (the consequences of) its behaviour [47], it can detect surprises – (e.g., the fact that an expected cue or reward was not observed) and contextual changes – and thus transfer control. It may be thus the failure to appropriately monitor and update contextual information, and not just the use of a habit, that underwrites behavioural inflexibility.

Another implication of our proposal is that deliberative and habitual choices are not learned in parallel, as assumed by the multicontroller view [10], but rather, the latter derives from (caching) the former. In other words, deliberative strategies are acquired first and scaffold the acquisition of habitual strategies. For example, habitual strategies may derive from a sort of “compression” or “caching” of deliberative strategies (e.g., by chunking action sequences) that entail a more parsimonious use of cognitive resources while preserving accuracy – at least when the agent has no residual uncertainty about the environment [11], [12], [23], [49], [60], [61]. This leads us to the next point, which concerns the relations between our scheme and Bayesian model selection or averaging.

### 5.2 Relations with Bayesian model selection (or averaging)

As discussed above most reinforcement learning theories assume that model-based and model-free controllers learn in parallel, our formulation is more related to the alternative view that habitual policies derive from the progressive simplification or compression (via caching, chunking or other methods) of a deliberative controller [12], [23], [60]. The general idea is that simplifying the computations (or reducing the generative model) required for the full active inference is yet another way to minimize free energy. In the present study, the simplification consists in caching policy probabilities. However, there are other alternative ways to simplify active inference. A previous simulation has shown that an active inference agent can progressively acquire state-action (or stimulus-response) policies that can be directly activated by stimuli rather than by internal inferential processes [23]. Another possibility consists in using minimal predictive controllers that are much reduced compared to the (relatively) sophisticated generative model considered in our simulations, but yet are sufficient to guide simple forms of adaptive behaviour [18]. Interestingly, these methods to simplify inference have close connections with Bayesian model selection (or averaging).

Indeed, there is a close connection between policy selection and Bayesian model selection (or averaging). This follows from the fact that the expected free energy (**G**_*π*_) value is the expected log model evidence or marginal likelihood, under each policy. Therefore, in active inference, selecting a policy corresponds to Bayesian model selection, where the quality of a policy becomes the evidence for that policy, expected under current beliefs about the state of the world. This means that there is a graceful link between policy selection, the balance between goal directed and habitual behaviour and the notion of Bayesian model selection or averaging in determining the best action. Previous work has explored the idea that control can be allocated amongst various internal models by weighting the accuracy and complexity of the candidate controllers [18]. The balance between full deliberation and habits explored here can be described in an analogous way. However, here we have considered the possibility that the simplest controller is not based on an internal generative model as in [18] but potentially a model-free construct such as a list of (cached) policy probabilities under different contexts (consider Panels E in Fig. 4, Fig. 5, Fig. 6).

Furthermore, it is possible to cast the model-selection process as a serial evaluation process, which only considers the more complex (e.g., deliberative) model if it has any chance to improve over the simpler model (e.g., in the cache), given knowledge of the current state. In other words, it is only when the simpler model is judged insufficient that a more complex model is considered, thus saving on computational resources [62]. In slightly more formal terms, one can imagine that the agent has a priori knowledge of the ratio of complexity of the two models (say, the simpler model has 2/3 the complexity of the more complex model). This would imply that the agent could automatically set a threshold of accuracy for engaging the simpler model (in this case, 67%). Indeed, if the ratio of the accuracies is 2/3 in favour of the simpler controller, and the simpler controller has 67% accuracy, its model selection (accuracy vs. complexity) score would be higher of the more complex model, even if this model has 100% accuracy (because 67/100 > 2/3). Analogously, if one considers a hierarchical active inference architecture, one can imagine that simpler controllers are lower in the hierarchy and are able to steer action when their accuracy or precision is sufficiently high. When this is not the case, more complex controllers that are higher in the hierarchy are also engaged and contextualize action selection [54], [60]. One can also look at this model selection problem the other way around, and consider that deliberative processing may be engaged when possible, but there may be conditions (e.g., dual tasks or mental fatigue) that prevent doing so [50]. In all cases, the model selection problem can be modelled in terms of a cost-benefit trade-off between resources required (e.g., complexity and/or its proxy that we measured used here: computational time) and accuracy of deliberative or habitual schemes.

A more abstract take on this issue derives from the imperative to minimise the path or time integral of free energy. If we consider natural selection as a form of (hierarchical) Bayesian model selection, then computational efficiency becomes operationally important – and a key determinant of the time integral of free energy [21]. This means that agents or phenotypes that can minimise free energy quickly will be selected over agents that do not. Technically, this is just a restatement of Hamilton’s principle of least action, because the time integral of free energy is a Hamiltonian action.

### 5.3 Relations with neurophysiology

It is widely assumed that, at the neurophysiological level, deliberate and habitual control might use partially different neuronal circuits, engaging for example dorsolateral striatum for habits and dorsomedial striatum for deliberation and forward search [15] (but see [41] for evidence for a more integrative view). Habitisation has been associated with the transfer of control to the dorsolateral striatum, where neural representations of habits have been reported that bear an important similarity with our simulations. When a rat performs a new task, these neurons only fire at reward locations; but this activity “shifts” back (gradually) to the start location when the task is routinised [34]. This behaviour is similar to changes in policy values at the start location (states 1 and 4 in Fig. 4, Fig. 5, Fig. 6). When a habit has been selected, these neurons fire both at start and goal location – a phenomenon that has been termed “task bracketing” [80]. While firing at start locations may be linked to action selection, firing at the end of the trial might reflect policy value or contextual updates – of the kind that characterise the schemes described above.

The neuronal underpinnings of deliberate and habitual choices extend beyond the striatal areas described above and include other subcortical and cortical networks [15], [52], [63], [68]. For example, several studies have showed that lesions to orbitofrontal cortex (OFC) or basolateral amygdala render animals insensitive to reinforcer devaluation and promote behavioural inflexibility [4], [31], [65], [66]. As discussed above — in the schemes proposed here — severe forms of behavioural inflexibility may stem from the failure of monitoring contextual changes or of updating state estimations appropriately. It is possible to speculate that lesions to structures like the OFC and the amygdala may compromise these functions, thus making animals unable to adapt to changes in task and reward contingencies – and ultimately to deploy flexible goal-directed behaviour. These ideas remain to be fully tested in future studies.

Another open question is whether a central arbitrator (possibly in frontal cortices [40], [69]) monitors the opportunity to engage deliberative mechanisms; using something analogous to the simple threshold-passing method described here. A possible alternative is that the balance between deliberate and habitual behaviour depends on a more hierarchical and distributed architecture, in which higher hierarchical layers (encoding more deliberative mechanisms) contextualize lower hierarchical layers (encoding habitual patterns that can be triggered by environmental cues) – but the latter can become relatively insensible to the former when they acquire sufficient precision [60], [61]. Either way, at some point the neural system would engage model-based planning and the (serial) evaluation of candidate policies, possibly in prefrontal areas and – at least in spatial tasks – the hippocampus [8], [57], [59], [62], [64], [69]. Note that the deliberative system can be engaged off-line, too; for example, to support memory consolidation and to train the habitual system [39], [56], [79]. This would allow to cache policy expectations even before an actual choice has to be made (e.g., during sleep or between decision episodes rather than prior to an actual decision) – hence effectively employing (costly) model-based computations to form cached memories for future use, rather than for (or in addition to) on-line decision and planning. This mechanism has been often linked to internally generated hippocampal dynamics and the “replay” of experience [13], [20], [57], [75].

Future studies are required that test these ideas, by assessing the conditions that promote the engagement deliberative processing; the transfer of control from deliberative to habitual processing, and vice versa; and the relative importance of model-based computations for on-line and off-line uses.

## Declaration of interest

None.

## Appendix A Mathematical formulation of Active Inference

Active Inference in discrete time and with discrete states can be described as a partially observable Markov decision process or POMDP. It is defined as a tuple (*P, Q, R, S, A, U*, Ω) where

• Ω is a finite set of observations *o*
• *A* is a finite set of action *a*
• *S* is a finite set of hidden states *s*
• *U* is a finite set of control states *u*
• *R* is a probability distribution over $\tilde{o}\in \Omega ,$ $\tilde{s}\in S$ and $\tilde{a}\in A$ — respectively sequences over time of observations, hidden states and actions — generated by a *generative process* and such that:
  $$\begin{array}{ccc} R(\tilde{o},\tilde{s},\tilde{a}) & = & \mathrm{Pr}(\left\{o^{\left(0\right)},\ldots ,o^{\left(t\right)}\right\}=\tilde{o},\left\{s^{\left(0\right)},\ldots ,s^{\left(t\right)}\right\}=\tilde{s},\\ & & \left\{a^{\left(0\right)},\ldots ,a^{\left(t-1\right)}\right\}=\tilde{a}) \end{array}$$
• *P*, the *generative model*, is a probability distribution over $\tilde{o}\in \Omega ,$ $\tilde{s}\in S,$ policies *π_j_*, j=1…,K — where *π_j_* returns a sequence of control states $\left\{u^{\left(t\right)},\ldots ,u^{\left(T\right)}\right\}\equiv \left\{\pi_{j}^{\left(t\right)},\ldots ,\pi_{j}^{\left(T\right)}\right\}$ — and parameters ***θ***, such that:
  $$\begin{array}{ccc} P(\tilde{o},\tilde{s},\pi_{j},\theta ) & = & \mathrm{Pr}(\left\{o^{\left(0\right)},\ldots ,o^{\left(T\right)}\right\}=\tilde{o},\left\{s^{\left(0\right)},\ldots ,s^{\left(T\right)}\right\}=\tilde{s},\\ & & \left\{u^{\left(0\right)},\ldots ,u^{\left(T\right)}\right\}=\pi_{j},\theta =\theta_{\psi }) \end{array}$$
  with parameters *θ*;
• *Q* is an approximate posterior over hidden and control states and a variable γ∈R denoted as *precision*, such that:
  $$Q(\tilde{s},\pi_{j},\gamma |\mu )=\mathrm{Pr}(\left\{s^{\left(0\right)},\ldots ,s^{\left(T\right)}\right\}=\tilde{s},\left\{u^{\left(t\right)},\ldots ,u^{\left(T\right)}\right\}=\pi_{j})$$
  with sufficient statistics (posterior expectation) $\hat{\mu }=\left(\hat{s},\hat{\pi },\hat{\gamma }\right)$.

Active inference rest on a continuous interplay among three probability distributions: the former (*generative process*) describing the external environment and its dynamics, and the latter two (*generative model* and *approximate posterior* over hidden states and parameters) describing internal dynamics of an agent. The *generative process* describes how transitions between hidden states of the external environment generate the observed outcomes through actions. In turn, these actions correspond to control states which are determined based on the agent’s beliefs about the next state(s). These beliefs are generated within the agent’s *generative model* that describes how the agent represents the environment and his exchanges with it. However, to circumvent the intractability of exact inference, the beliefs are expectations from an *approximate posterior* of the full generative model; as explained below.

### A.1 The generative model

In term of Markov decision process, the generative model, with parameters θ={A,B,C,D,α,β} can be dynamically expressed as:

(A.1) $$\begin{array}{ccc} & & P\left(\tilde{o},\tilde{s},\pi_{j},\gamma \right)=P\left(\pi_{j}\right)P\left(\gamma \right)P\left(\tilde{o}|\tilde{s}\right)P\left(\tilde{s}|\pi_{j}\right)\\ & & \mathrm{where}\\ & & P\left(\tilde{o}|\tilde{s}\right)=\prod_{i=0}^{t}P\left(o^{\left(i\right)}|s^{\left(i\right)}\right)\\ & & P\left(\tilde{s}|\pi_{j}\right)=P\left(s^{\left(0\right)}\right)\prod_{i=1}^{t}P\left(s^{\left(i\right)}|s^{\left(i-1\right)},\pi_{j}\right) \end{array}$$

The first equality expresses the model as joint distribution that comprises conditional densities and empirical priors. The two expressions $P(\tilde{o}|\tilde{s})$ and $P(\tilde{s}|\pi_{j})$ establish that observations exclusively depend upon the co-occurrent hidden states; and that dependencies between successive hidden states are Markovian, respectively.

A generative model can be parameterised in a general way as follows:

(A.2a) $$P(o^{\left(t\right)}|s^{\left(t\right)})=A$$

(A.2b) $$P(s^{\left(t+1\right)}|s^{\left(t\right)},\pi_{j})=B\left(u^{\left(t\right)}=\pi_{j}^{\left(t\right)}\right)$$

(A.2c) $$\;P(o^{\left(\tau \right)})=C$$

(A.2d) $$P(s^{0})=D$$

(A.2e) $$P(\pi_{j})=\sigma (\gamma \cdot G_{\pi })$$

(A.2f) P(γ)∼Γ(α,β)

Accordingly, **A** encodes the likelihood of observations given the state (Eq. A.2a) while the prior distributions over initial state and future outcomes are encoded in Eqs. (A.2d) and (A.2c), respectively with matrices **D** and **C**. State transitions are encoded with **B** and depend on policy (Eq. A.2b) whose distribution (Eq. A.2e) is attained from a softmax function *σ*( · ) of the inner product ($A\cdot B=A^{T}B$) between its expected free energy **G**_*π*_ and the precision *γ* sampled from a Γ distribution with parameters *α* and *β* (Eq. A.2f).

The quantity expressed with **G**_*π*_ deserves more attention. It can be considered as the *quality* of a generic policy *π_j_* and substantially plays the role of (prior) beliefs over the policy, with respect to the current state and the preferred outcomes. These beliefs rest on (preferred) outcomes in the future, because these beliefs determine action and action determines subsequent outcomes. This means that policies should, a priori, minimise the free energy of beliefs about the future – i.e., an expected free energy. Formally, it is possible determine this quality value by using with the path integral^2^ (from the current at time *t* to the final state at time *T*) of free energy expected under the policy *π_j_* [26]:

(A.3) $$G_{\pi }=\sum_{\tau =t}^{T}G\left(\pi_{j},\tau \right)$$

where

(A.4) $$G\left(\pi_{j},\tau \right)=-E_{\tilde{Q}}\left[H\left[P(o^{\left(\tau \right)}|s^{\left(\tau \right)})\right]\right]-D_{\mathrm{KL}}\left[Q\left(o^{\left(\tau \right)}|\pi_{j}\right)∥P\left(o^{\left(\tau \right)}\right)\right]$$

and $\tilde{Q}=Q\left(o^{\left(\tau \right)},s^{\left(\tau \right)}|\pi_{j}\right)$. The above expression can be expressed in term of the generative model and taking a more compact and simpler form:

(A.5) $$\begin{array}{ccc} & & G_{\pi }=\sum_{\tau =t}^{T}\left(H\cdot {\hat{s}}_{\pi }^{\left(\tau \right)}-\left(\ln{\hat{o}}_{\pi }^{\left(\tau \right)}-lnP\left(o^{\left(\tau \right)}\right)\right)\cdot {\hat{o}}_{\pi }^{\left(\tau \right)}\right)\\ & & {\hat{s}}_{\pi }^{\left(\tau \right)}=B\left(u^{\left(\tau \right)}=\pi_{j}^{\left(\tau \right)}\right)\cdot {\hat{s}}^{\left(\tau \right)}\\ & & {\hat{o}}_{\pi }^{\left(\tau \right)}=A\cdot {\hat{s}}_{\pi }^{\left(\tau \right)}\\ & & lnP\left(o^{\left(\tau \right)}\right)=\ln C_{\tau }\\ & & H=-(A\times \ln A) \end{array}$$

where “ × ” denotes a product element-by-element. Note that two terms in Eq. (A.5) represent *ambiguity* (i.e., uncertainty about outcomes, in relation to the state of the world) and *risk* (i.e., relative entropy or uncertainty about outcomes, in relation to preferences), respectively; where utility *lnP*(*o_τ_*) is a vector of preferences over future outcomes.

### A.2 The approximate posterior

In general, Bayesian inference using the full generative model is intractable. To circumvent this problem, active inference appeals to approximate (variational) inference using an approximate posterior rather than the full generative model.

Therefore, after having specified the shape of the generative model, it is necessary to specify its approximate posterior $Q(\tilde{s},\pi ,\gamma |\mu )$ – for which we need to find the sufficient statistics $\mu =\left(\hat{s},\hat{\pi },\hat{\gamma }\right)\in R^{+}$ that minimise free energy. The role of the sufficient statistics *μ* is to allow the factorisation of the approximate posterior into marginal distributions that reduce the size of the state space (thus render inference tractable) by replacing posterior dependencies among hidden variables.

By exploiting the Markovian dependencies among successive states, we can assume that, at each time *t*, the approximate posterior can be factorised over past hidden states, future control and precision:

(A.6) $$Q\left(\hat{s},\hat{\pi },\gamma |\mu \right)=Q\left(s^{\left(0\right)}|{\hat{s}}^{\left(0\right)}\right)\;\cdots \;Q\left(s^{\left(t\right)}|{\hat{s}}^{\left(t\right)}\right)Q\left(u^{\left(t\right)},\cdots ,u^{\left(T\right)}|\hat{\pi }\right)Q\left(\gamma |\hat{\gamma }\right)$$

where

$$\begin{array}{ccc} & & Q\left(s^{\left(t\right)}=i|{\hat{s}}^{\left(t\right)}\right)={\hat{s}}_{i}^{\left(t\right)}\;\mathrm{with}\;\sum_{i}{\hat{s}}_{i}^{\left(t\right)}=1\\ & & Q\left(\tilde{u}\equiv \pi_{k}|\hat{\pi }\right)={\hat{\pi }}_{k}\;\mathrm{with}\;\sum_{k}{\hat{\pi }}_{k}=1\\ & & Q\left(\gamma |\hat{\gamma }\right)∼\Gamma \left(\alpha ,\hat{\beta }=\alpha /\hat{\gamma }\right) \end{array}$$

Eq. (A.6) represents the *mean field assumption* by means of which the approximate posterior *Q* can factorise over its parameters [35]. Note that because we have used the Markovian property of the generative model, this is not an approximation – except for precision, given the existence of conditional dependencies between precision and hidden states.

Equipped with the generative model (Eq. A.1) and the approximate posterior with the mean field assumption (Eq. A.6), it is possible to minimise variational free energy and solve the Bayesian inference.

### A.3 Free energy and variational Bayes inference

Active inference rests on free energy minimisation with respect to sufficient statistics (expectations) of the approximate posterior about hidden states, control states (policies) and precision. More precisely, active inference assumes that, given the free energy:

(A.7) $$F\left(\tilde{o},\mu \right)=E_{Q}\left[-\ln P(\tilde{o},\tilde{s},\pi ,\gamma )\right]-H\left[Q(\tilde{s},\pi ,\gamma |\mu )\right]$$

there exist sufficient statistics $\hat{\mu }$ that minimise *F*:

$${\hat{\mu }}_{t}=\arg\min_{\mu }F\left(\tilde{o},\mu \right)$$

such that the ensuing beliefs about control states comprised in the policies prescribe current action:

$$Pr\left(a^{\left(t\right)}=u^{\left(t\right)}\right)=Q\left(u^{\left(t\right)}|{\hat{\mu }}^{\left(t\right)}\right)$$

Bayesian inference (which consists in calculating posterior from prior beliefs) thus turns into an optimisation problem – where we need to find out the values ${\hat{\mu }}_{t}$ that minimise the objective function represented in Eq. (A.7).

Sufficient statistics, and implicitly Bayesian estimations of hidden variables, are obtained by iterating the following updating equations (see Appendix B of [26] for details):

(A.8) $$\begin{array}{ccc} {\hat{s}}^{\left(t\right)} & \approx & \sigma \left(\ln A\cdot o^{\left(t\right)}+\ln\left(B\left(a^{\left(t-1\right)}\right)\cdot {\hat{s}}^{\left(t-1\right)}\right)\right)\\ \hat{\pi } & = & \sigma (\hat{\gamma }\cdot G_{\pi })\\ \hat{\gamma } & = & \frac{\alpha }{\beta -G_{\hat{\pi }}} \end{array}$$

The first equation updates expectations about hidden states and corresponds to perceptual inference or state estimation (note that the dependency of value on expected states – or optimism – bias has been ignored for simplicity, given it is not numerically relevant).

The second update is a softmax function of the value of each policy, where the sensitivity parameter is an increasing function of expected value. This means that the sensitivity, or inverse temperature, that determines the precision with which a policy is selected, increases with the expected value of those policies. This essentially renders action selection more deterministic when better policies are available.

The third update optimises expected precision. As can be easily appreciated, if an observation increases the expected value of the policies entertained by an agent, then expected precision increases (i.e., temperature decreases) and the agent is implicitly more confident in selecting the next action. Minimization of free energy appeals to the iteration of these three updates are – which have also nice correspondences in terms of brain dynamics, see [24].

## Footnotes

1 Note that this scheme uses the policy probabilities of states 17 and 40 (not states 1 and 4) to decide whether or not to habitise (because, as explained in the main text, these are the max values in each context). Here, we show the probabilities of states 1 and 4, for consistency with the two subsequent schemes and because the relative probabilities of the policies are very similar in states 1 and 17 (as well as in states 4 and 40); although of course the absolute values are not.
